# Facial speech processing in children with and without dyslexia

**DOI:** 10.1007/s11881-021-00231-3

**Published:** 2021-06-11

**Authors:** Martyna A. Galazka, Nouchine Hadjikhani, Maria Sundqvist, Jakob Åsberg Johnels

**Affiliations:** 1 Gillberg Neuropsychiatry Center, Institute of Neuroscience and Physiology, University of Gothenburg, Gothenburg, Sweden; 2 Harvard Medical School/MGH/MIT, Athinoula A. Martinos Center for Biomedical Imaging, Boston, MA USA; 3 Department of Education and Special Education, University of Gothenburg, Gothenburg, Sweden; 4 Section of Speech and Language Pathology, Institute of Neuroscience and Physiology, University of Gothenburg, Gothenburg, Sweden

**Keywords:** Articulation, Audiovisual, Dyslexia, Eye tracking, Facial speech, Reading

## Abstract

What role does the presence of facial speech play for children with dyslexia? Current literature proposes two distinctive claims. One claim states that children with dyslexia make less use of visual information from the mouth during speech processing due to a deficit in recruitment of audiovisual areas. An opposing claim suggests that children with dyslexia are in fact *reliant* on such information in order to compensate for auditory/phonological impairments. The current paper aims at directly testing these contrasting hypotheses (here referred to as “mouth insensitivity” versus “mouth reliance”) in school-age children with and without dyslexia, matched on age and listening comprehension. Using eye tracking, in Study 1, we examined how children look at the mouth across conditions varying in speech processing demands. The results did not indicate significant group differences in looking at the mouth. However, correlation analyses suggest potentially important distinctions within the dyslexia group: those children with dyslexia who are better readers attended more to the mouth while presented with a person’s face in a phonologically demanding condition. In Study 2, we examined whether the presence of facial speech cues is functionally beneficial when a child is encoding written words. The results indicated lack of overall group differences on the task, although those with less severe reading problems in the dyslexia group were more accurate when reading words that were presented with articulatory facial speech cues. Collectively, our results suggest that children with dyslexia differ in their “mouth reliance” versus “mouth insensitivity,” a profile that seems to be related to the severity of their reading problems.

**Supplementary Information:**

The online version contains supplementary material available at 10.1007/s11881-021-00231-3.

## Introduction

Developmental dyslexia refers to behaviorally defined difficulties in developing fluent and accurate word decoding which cannot be attributed to either low mental or chronological age or sensory-neurological disorders (American Psychological Association, 2013; Lyon, 1995; Snowling et al., 2020; Vellutino et al., 2004). Poor word reading negatively impacts children’s reading comprehension, increases the risk of school failure (Grizzle, 2007; Lyytinen et al., 2015; Nordström et al., 2016; Vellutino & Fletcher, 2005), and is linked to poorer mental wellbeing (Riddick et al., 1999; Russell et al., 2015). A large body of research suggests that an important underlying problem in dyslexia lies in the individual’s phonology, that is, the ability to process the sound structure of language, making it difficult to establish the links between letters and phonemes (Lyon, 1995; Snowling, 2001; Snowling et al., 2020). The use of facial speech cues (articulation) can facilitate phonological processing and perhaps, by extension, support the establishment of grapheme/phoneme associations. Here we examined how school children with and without dyslexia use facial speech during language perception.

Typical language development takes place within a rich audiovisual context, meaning that when children hear someone speak, they almost always simultaneously see synchronous facial patterns and changes in the movement of the speaker’s mouth (Kuhl & Meltzoff, 1982). Because of this frequent pairing, there are good reasons to assume that visual information during facial speech plays an important role in language development and language processing. Indeed, over the years, great amount of research has shown that the perception of speech in one modality is tightly connected to perception of information in the other (Green et al., 1991; Massaro & Cohen, 1983; McDonald et al., 2000; Skipper et al., 2009). In fact, seeing particular lip movement or mouth shape has been shown to activate the auditory cortex, even in the absence of auditory input (Calvert et al., 1997). Similarly, articulatory information from the lips and mouth shape has been shown to enhance phonetic category learning (Hirata & Kelly, 2010; Teinonen et al., 2008), meaning that for instance seeing a rounded mouth, even before any sound is made, allows the observer to rule out sounds that are visually incompatible with that particular shape (e.g., an /*e*/ sound).

Interestingly, spontaneous gaze behavior seems to correspond with increased use of the facial speech in language discrimination. Eye tracking research has shown that in children who are learning to speak, increased gaze toward the lower portions of the face, namely the mouth and lips, peaks during periods of increased language development (de Boisferon et al., 2018; Lewkowicz & Hansen-Tift, 2012). In turn, this tendency corresponds with higher expressive language and vocabulary size later in development (Young et al., 2009). One potential cause for increased mouth observation early in development is that it serves as a scaffolding mechanism for language development, by facilitating phonological tuning (Lewkowicz, 2014; Lewkowicz & Hansen-Tift, 2012; Magnotti & Beauchamp, 2017). Looking at the mouth is thus presumed to reflect a growing sensitivity to articulatory information in visual speech recognition (Thomas & Jordan, 2004; Yehia et al., 1998). However, the association between mouth gazing and language processing is complex and likely age dependent, because while looking at the mouth in infancy that has been suggested to longitudinally support language processing (Young et al., 2009), excessive attention to the mouth in preschool-aged children has been associated with language comprehension deficits (e.g., Åsberg Johnels et al., 2014; Hosozawa et al., 2012). For adults, seeing a speaker’s mouth, face, and head movement appears useful in a range of situations, perhaps especially when listening demands are high: for instance, in low or noisy environments (Lansing & McConkie, 2003; Munhall et al., 2004; Rosenblum et al., 1996; Vatikiotis-Bateson et al., 1998), when needing to discriminate between phonemes while learning a second language (Hirata & Kelly, 2010), while performing a difficult language detection task (Barenholtz et al., 2016), or when presented with a speaking face in a complex dynamic setting (Võ et al., 2012).

Given the difficulties that individuals with dyslexia have with phonological processing (Vellutino et al., 2004), facial speech perception in this group has gained some interest. Still, to date, the literature on the role and use of visual information during speech processing in this group is rather sparse, and the few studies that do exist seem to report curiously conflicting claims. One body of research has examined lip-reading capacities, with some reporting that dyslexic readers have deficits in the ability to benefit from the presence of lip-read words (van Laarhoven, Keetels, Schakel & Vroomen, 2018) and are worse at lip-reading compared to non-dyslexic controls (de Gelder & Vroomen, 1998), perhaps due to a deficit in the adequacy of phonological representations (Goswami, 2003). At the same time, other studies in school-aged children report no distinct differences between dyslexic and non-dyslexic readers in the identification of speech based on visual cues from talking faces alone or lip-reading but instead suggest a unique impairment in auditory categorization (Baart et al., 2012). Still others (Francisco et al., 2017a, b) find that for adult university students with dyslexia, lip-reading ability uniquely contributes to variance in phonological awareness, with those who score lower on phonological awareness (more severely impaired) being also better lip-readers. This finding seems to support the claim that increased reliance on visual speech may be a compensatory mechanism when processing auditory speech alone is problematic (Francisco et al., 2017a, b).

Another way of trying to examine the contribution auditory and visual information is to present congruent and incongruent facial speech, in which visual information (mouth making the /*b*/ sound) either matches or not the auditory input. Using this methodology, some reports attribute phonological difficulties in dyslexia to a distinct deficit in multisensory integration that make visual access to facial speech less salient and useful (Groen & Jesse, 2013; Hayes et al., 2003; Norrix et al., 2006; Ramirez & Mann, 2005; van Laarhoven et al., 2018). For instance, when passively observing videos of faces producing congruent or incongruent syllables, Rüsseler et al. (2018) found that individuals with dyslexia showed a *reduced* activity in the fusiform gyrus and occipital gyrus, indicating a deficit in extracting information from the face, although it is unclear which particular areas of the face were attended (see for instance Morris et al., 2007). Additionally, they report reduced activity in the superior temporal sulcus (STS), an area responsible for multimodal audiovisual processing. This pattern has been attributed to “a general impairment in the recruitment of audiovisual areas in dyslexia” (pg. 366). These conclusions are supported by other reports (Blau et al., 2009, 2010; Francisco et al., 2018; Kast et al., 2011; Ye et al., 2017). Similarly, when examining ERP signals in children with dyslexia during perception of audiovisual speech, a reduced enhancement of the amplitude of the mismatch negativity response (MMR) to bimodal compared with monomodal (visual only or auditory only) speech was noted, indicating that dyslexic children did not benefit from facial speech presentation to the same degree as their non-dyslexic peers (Schaadt et al., 2019; see also Rüsseler et al., 2015).

Contrary to Schaadt et al. (2019), one study (Pekkola et al., 2006) reported an *increase* rather than a reduction in activation of brain areas presumed to support speech in a dyslexic group when watching a movie of a person whose mouth movements did not correspond to heard auditory input. This activation co-varied with phonological processing abilities (worse phonological processing corresponded to increased activation), interpreted as reflecting “dyslexic readers heightened reliance on motor-articulatory and visual speech processing strategies, possibly as a compensatory mechanism to overcome linguistic perceptual difficulties” (pg. 804). Similarly, Schaadt et al. (2016) presented dyslexic and non-dyslexic 10-year-olds with a video recording of a speaker’s mouth that was silently pronouncing syllables. They found that non-dyslexic children displayed an increased posterior response to a sudden change in the “pronounced” syllable (known as the visual mismatch response (vMMR)) consistent with processing of visual input. Children with dyslexia, on the other hand, displayed increased anterior vMMR consistent with processing of auditory input, even when none was present. This effect was especially evident in children with severe phonological deficits. Here again, the authors interpreted the findings in terms of compensatory strategy, meaning that dyslexic children with the most severe phonological deficits recruit auditory processing mechanisms in anticipation of auditory input to support phonological processing. Specifically, they argue that “individuals with dyslexia use visual speech information in an attempt to compensate for their phonological deficit,” (pg. 1032) but at the same time, the authors acknowledge that further research is critical in order to examine whether this compensatory strategy is functional.

Taking into consideration findings and interpretations from these different studies, two sharply contrasting hypotheses can be formulated. The first is that children with dyslexia do not benefit from the presence of visual cues during facial speech processing, potentially due to a more general deficit in integrating the two modalities, referred to below as “mouth insensitivity.” The alternative possibility is that children with dyslexia use and indeed benefit from visual articulatory information as a way to compensate for their difficulties in auditory speech perception, referred to as “mouth reliance.” If the mouth reliance hypothesis is correct, the benefit of visual cues, in the form of visual articulations, will be evident within a learning context. Here, we performed two studies in an attempt to clarify these mechanisms. In so doing, we first assessed whether children with dyslexia are sensitive to the presence of visual cues by examining spontaneous attention to the mouth during speech perception. Then, through experimental manipulation, we examined whether the presence of visual cues is functionally beneficial in a phoneme-/grapheme-based word decoding task.

## Study 1

To date, there are no studies on how children with dyslexia, with well-known phonological processing difficulties, naturally scan faces during speech perception. This approach has been used in research on typically developing children (Lewkowicz & Hansen-Tift, 2012), as well as in other groups of children with language or communication disorders (Åsberg Johnels et al., 2014; Falck-Ytter et al., 2010; Irwin et al., 2021). A straightforward way of testing the facial speech processing capacities in dyslexia can be performed by evaluating whether dyslexic children look at a speaking mouth in the same manner as peers from a community sample who do not have reading difficulties or difficulties with phonological processing. Here, we presented school children, with and without diagnosed dyslexia, a video of a female speaker who was silent (silent face condition), telling short stories (ordinary speech condition), and was pronouncing nonsense words that the participants were instructed to repeat (nonword repetition condition). Across these three conditions, gaze patterns toward the mouth were calculated to determine dyslexic participants’ reliance on the mouth compared to a non-dyslexic group that were matched on age and listening comprehension. Compared to the silent face condition, we expect typically reading children without dyslexia to ramp up their mouth gazing during the ordinary speech condition and nonword repetition and especially the latter, which has been designed to be phonologically taxing. Keeping in mind the two proposed hypotheses discussed above, if children with dyslexia are “mouth insensitive,” we would expect similar looking times across the conditions. In addition, compared with community controls, one could hypothesize less gaze toward the mouth, particularly in conditions with ordinary speech and nonword repetition. If, however, they are reliant on the mouth during speech processing (“mouth reliance”), we would expect increased gaze to the mouth when processing language information that is phonologically more challenging. Finally, in considering several studies (de Gelder & Vroomen, 1998; Schaadt et al., 2016; Schaadt et al., 2019) that report associations between reading-related skills presumed to reflect facial speech processing, we will examine associations between reading-related measures and proportion of looking at the mouth within each group.

### Method

#### Ethics

All procedures were conducted in accordance with the Declaration of Helsinki 1964 (World Medical Association, 2001) and were approved by the local ethical committee (1090-17). Written consent from parents and verbal assent of child participants was obtained prior to testing.

#### Participants

A total of 46 Swedish-speaking children between the ages of 9–13 years were tested in the study. There are two reasons to focus on this age group: first, this is the age when the diagnosis of dyslexia is usually first considered in Sweden (where children start school at the age of 7), and second, during this time, word reading is expected to be fluent; indeed, the 3rd or 4th grade is often considered to represent a shift in emphasis in instruction from “learning to read” to “reading to learn.” Of the 46 participants, 3 were excluded due to technical difficulties with the eye tracker (n = 1), not being a native speaker (n = 1), and refusal to continue (n = 1). Of the remaining 43, 18 belonged to the dyslexia (DYS) group, and 25 comprised the community comparison group (CON). In Sweden, dyslexia or “specific reading disorder” is diagnosed according to the ICD-10 (World Health Organization, 1992) typically with supporting information regarding phonological impairment and normal listening comprehension in line with a widely accepted working definition of dyslexia from the International Dyslexia Association (cf., Lyon, 1995). Hence, we recruited individuals with word reading and phonological problems but not general language disorder. Fifteen of the 18 children with dyslexia (DYS) were recruited from the speech-language pathology clinic where they received their diagnosis, while another two cases had received their diagnosis by a separate qualified clinician^1^. As part of their diagnostic assessments, children received a general health check to rule out hearing problems or any neurological or sensory abnormalities that would interfere with hearing or reading abilities.

Participants in the comparison group (CON) were recruited from local elementary schools and were matched with the dyslexia group on listening comprehension, age, and gender. None of the children had hearing problems according to parental reports, and none had Swedish as a second language. All children in the CON group and all but one in the DYS group were right-handed. All children participated in both Experiments 1 and 2 in the same session, in that order.

Parents of all participating children completed the Strengths and Difficulties Questionnaire (SDQ; Muris et al., 2003) which measures psychopathological symptoms in children. This scale is commonly used to screen for “comorbid” neurodevelopmental and mental health difficulties in dyslexia (Russell et al., 2015). In our sample, we did not exclude children scoring high on the SDQ since this would clearly affect the representativeness of the dyslexia sample. Following Hulme and Snowling (2013), we do however examine how comorbid symptoms according to SDQ relate to the main variables of interest.

### Measures

#### Psychoeducational assessments

##### Word reading

Participants’ word-reading efficiency was measured using the Swedish adaptation of the Test of Word Reading Efficiency (TOWRE), renamed LäSt (Elwer, 2015; Torgesen et al., 1999). This test is designed to quickly assess two kinds of word-reading skills critical in the development of the overall reading ability: the ability to quickly and accurately sound out words that participants have never encountered (nonword subscale) and the ability to quickly and accurately recognize familiar words (word subscale). In the assessment, participants are asked to read out loud as many single words as possible in 45 seconds from two lists/subscales. Scores from the lists are added to create final score. A test-retest reliability of .97 is reported in the Swedish manual (Elwer, 2015). Scores are expressed as either raw scores or in age-adjusted stanine scores (around a normative mean of 5, SD = 2). In order not to lose information and cause restriction of range, we use the raw scores in the analyses and highlight any associations with age. We do report the mean stanine scores for descriptive purposes.

##### Phonological processing

Phonological processing was assessed in all children using a subscale of the NEPSY Assessment (Korkman, 1998). In this assessment, children had to either omit parts of the word (e.g., omit “/dum/” in the word *dumhet*) or substitute parts of the word for another (e.g., in the word *flicka*, substitute the “/fl/” sound for “/br/” sound). Raw scores and age-adjusted z-scores were calculated based on normative means and SD reported in the manual.

##### Listening comprehension

This was assessed using the text comprehension subtest from the Swedish translation of the Clinical Evaluation of the Language Fundamentals–IV, *CELF-4* (Semel et al., 2004), which is an instrument used for identifying and diagnosing disorders in language performance (test-retest reliability is between .70 and .90). Scores are reported as raw scores and as scaled scores around a normative mean of 10 (SD = 3).

#### Apparatus

Gaze measures was collected using Tobii X2-30 (Tobii Technology Inc., Stockholm, Sweden), which records near-infrared reflections of both eyes at 30 Hz as the subject watches an integrated 17-in (33.7 × 27 cm) monitor at approx. 60-cm distance. A 9-point calibration procedure was performed once prior to the experiment in which an expanding and contracting ball is shown at nine locations on the screen. If the calibration indicated inadequate data, the calibration procedure was repeated until data was collected for all points. Lenovo ThinkPad with intel Core i7 vPro laptop with in-built loudspeakers was used in all testing. The iMotions (iMotions A/S, Copenhagen, Denmark) software was used for recording of eye gaze.

#### Facial speech eye tracking experiment

**Fig. 1:**
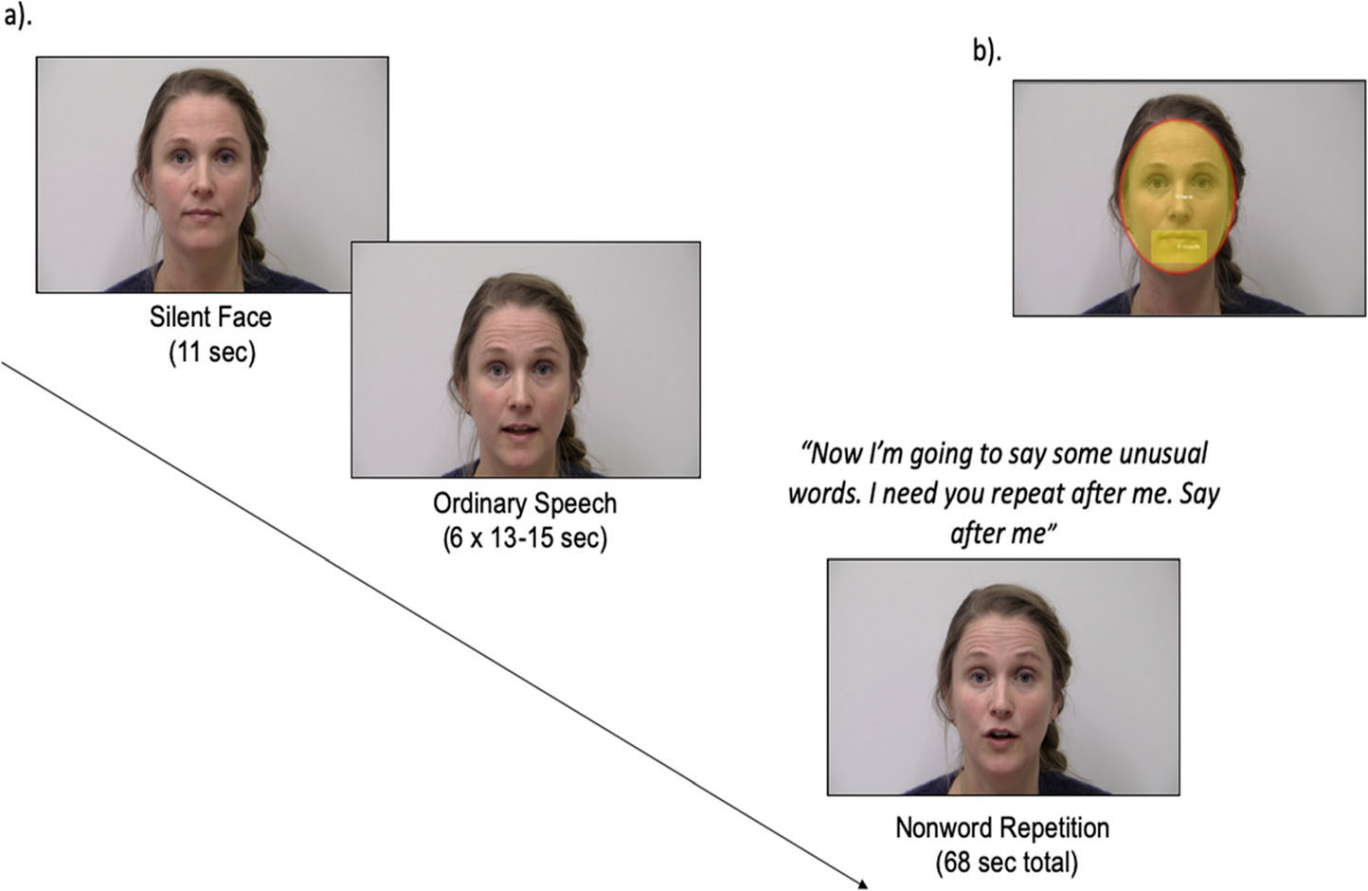
**a** Facial speech eye tracking experiment with silent face condition, ordinary speech condition, and nonword repetition condition. **b** Mouth and face AOIs

All participants were presented a video of a female actor across three speech conditions: silent face condition, ordinary speech condition, and nonword repetition condition (Fig. 1). In all conditions, the actor was looking directly at the camera and had a neutral facial expression.

##### Silent face condition

In the silent face condition, the female actor was silent and was generally not moving other than having naturally occurring facial movements such as eye blinks. The participants were instructed to simply observe the video. This condition lasted for 11 s.

##### Ordinary speech condition

Following the silent face condition, the participants observed the actor tell six 3-sentence short stories. Each story lasted between 13 and 15 s and was preceded and followed by 2 s of silence. Participants were simply instructed to observe the videos.

##### Nonword repetition condition

At the start of the video, the participants were instructed: “*Now I’m going to say some unusual words. I need you to repeat after me. Say after me*.” Next, the participants watched the screen as the female speaker said 9 nonwords that varied in length between 2 and 4 syllables. After each nonword, the actor was silent for approximately 5 s allowing participants to repeat what they heard. It is important to note that while the nonword repetition was meant to be phonologically challenging compared with the other conditions, the task was not designed to be sensitive to individual differences; almost all nonwords were correctly repeated by participants in both groups.

For a complete list of stories used in the ordinary speech condition and nonwords used in the nonword repetition condition, see Supplementary Materials.

### Data analysis

Following data collection, eye gaze recordings were exported from iMotions platform (iMotions A/S, Copenhagen, Denmark) and analyzed using Time Studio (Version 3.18; timestudioproject.com; Nyström et al., 2016), a MATLAB-based open access analysis tool specifically designed for analyzing time series data.

The exported data were examined for total fixations within specified areas of interest (henceforth, AOIs). Two AOIs were defined for the analysis: one around the speaker’s face (face AOI) and the second around the speaker’s mouth (mouth AOI). The face AOI was an elliptical shape encompassing the speakers face from the top of her forehead, excluding her hair, to the bottom of the chin and between the two ears, measuring 7.54 horizontal by 8.26 vertical visual degrees (440 × 480 pixels). The mouth AOI was a rectangle measuring 3.43 horizontal by 1.72 vertical visual degrees (200 × 100 pixels) (see Fig. 1b). Importantly, the AOIs used for this analysis were moving according to the position of the object in the video, and so the slight movement of the actor during speech conditions did not influence the placement of the AOIs. The exact parameters used for the analysis can be downloaded using uwid ts-aa9-872 from within the Time Studio program. Statistical analysis was performed using SPSS (version 27).

### Statistical analyses

In terms of statistical analysis, we first compared the two groups (DYS and CON) on the standardized measures of reading ability (LäSt; words and nonwords subscales), phonological processing (NEPSY), listening comprehension (CELF-4), as well as behavior (SDQ) using independent samples t-tests. Clearly the topic of “statistical significance” is contested in current theorizing, and several dominant voices in the field argue against the usage of p-based inferential language (Lakens et al., 2018). Since many of us are used to communicating in terms of p-values, we use p-based reasoning but also focus on clear illustration of results, of groups as well as individuals, and effect sizes for communicating the findings.

In the main statistical analysis, we examined mouth viewing as a dependent variable across three conditions in the two groups (DYS, CON). For each trial, *proportion of looking at the mouth* was computed by dividing the total fixations in milliseconds on the area around the actor’s mouth by the total fixations on the actor’s face, which were then averaged across trials for each condition. We used proportions of looking, rather than total fixations, in order to account for the different trial durations across the three conditions.

Finally, in order to examine whether correlations could be found within groups between the proportion of mouth looking and reading ability, we performed (non-parametric) analyses in each group separately, in order to reduce the impact of any outliers in the data set, that can otherwise affect results in small *n* studies. The significance level was set to *p* < .05 for 2-tailed tests. Because specific a priori hypotheses were tested on the most critical contrast, we did not use Bonferroni corrections.

In terms of interpretation, we focused on the magnitude of the correlations and on effects size, according to the conventional sizes of the *r* values proposed by Cohen (1988) for small, medium, and large effects to be .10, .30, and .50, respectively. In much the same way, using Cohen (1988), we define small (*η*^2^ = 0.01), medium (*η*^2^ = 0.06), and large (*η*^2^ = 0.14) effects for the between-group analysis.

## Results and discussion

Participants’ demographic and clinical characteristics are presented in Table 1. As expected, the two groups differed significantly on the measures of reading ability (LäSt; words and nonwords subscales) and phonological processing (awareness; NEPSY), with the DYS group scoring low, while the CON group scored very close to normative levels. There was also a significant difference in the SDQ total scores, with parents of DYS children reporting higher behavioral problems compared to the parents of children in the CON group. By contrast, the two groups were matched on listening comprehension, with both groups, on average, scoring within the age-adequate range according to population norms. Hence, as a group, the DYS readers displayed the pattern of poor word/nonword reading, poor phonological processing, but typical listening comprehension, which is typical for individuals receiving a dyslexia diagnosis.

**Table 1:** Participant’s demographic and clinical information

		DYS (N = 18)		CON (N = 25)		Independent samples t-test or Pearson’s Chi-square	
	Subscale	M (SD)	Range	M (SD)	Range	t (df)/χ^2^(df)	p-values
Age		10.61 (1.1)	9–13	10.96 (0.5)	10–12	9.25 (4)	0.055
Gender		9 boys; 9 girls		8 boys, 17 girls		1.42 (1)	0.234
Single word reading (LäSt)	Words	70.2 (29.6) ± 1.89 (1.32)^a^	31–145	132.7 (17.6) ± 4.75 (2.00)^a^	101–163	−8.66 (41)	<.001***
						−5.56 (39.46)	<.001***
	Nonwords	37.4 (12.9) ± 2.11 (1.08)^a^	20–64	76.3 (18.3) ± 5.04 (2.11)^a^	39–106	−7.75 (41)	<.001***
						−5.94 (37.58)	<.001***
Phonological processing (NEPSY)		28.7 (5.2) ± −1.03 (1.28)^b^	15–21	33.2 (1.8) ± −.013 (.44)^b^	29–36	−3.5 (19.8)	0.002**
Listening comprehension (CELF-4)		12.4 (1.7) ± 9.94 (2.55)^c^	9–15	12 (1.5) ± 9.16 (1.88)^c^	9–14	−3.24 (19.85)	0.004**
						.789 (41)	0.435
SDQ	Total	19.72 (6.04)	11–32	14.4 (5.1)	9–30	1.16 (41)	0.253
						2.97 (37)	0.005**

±Raw scores

^a^Stanine scores (normative mean = 5, std. dev. = 2)

^b^z-scores (normative mean = 0, std. dev. = 1)

^c^Scaled scores (normative mean = 10, std. dev. = 3

**p ≤ 0.01

***p ≤ 0.001

**Fig. 2:**
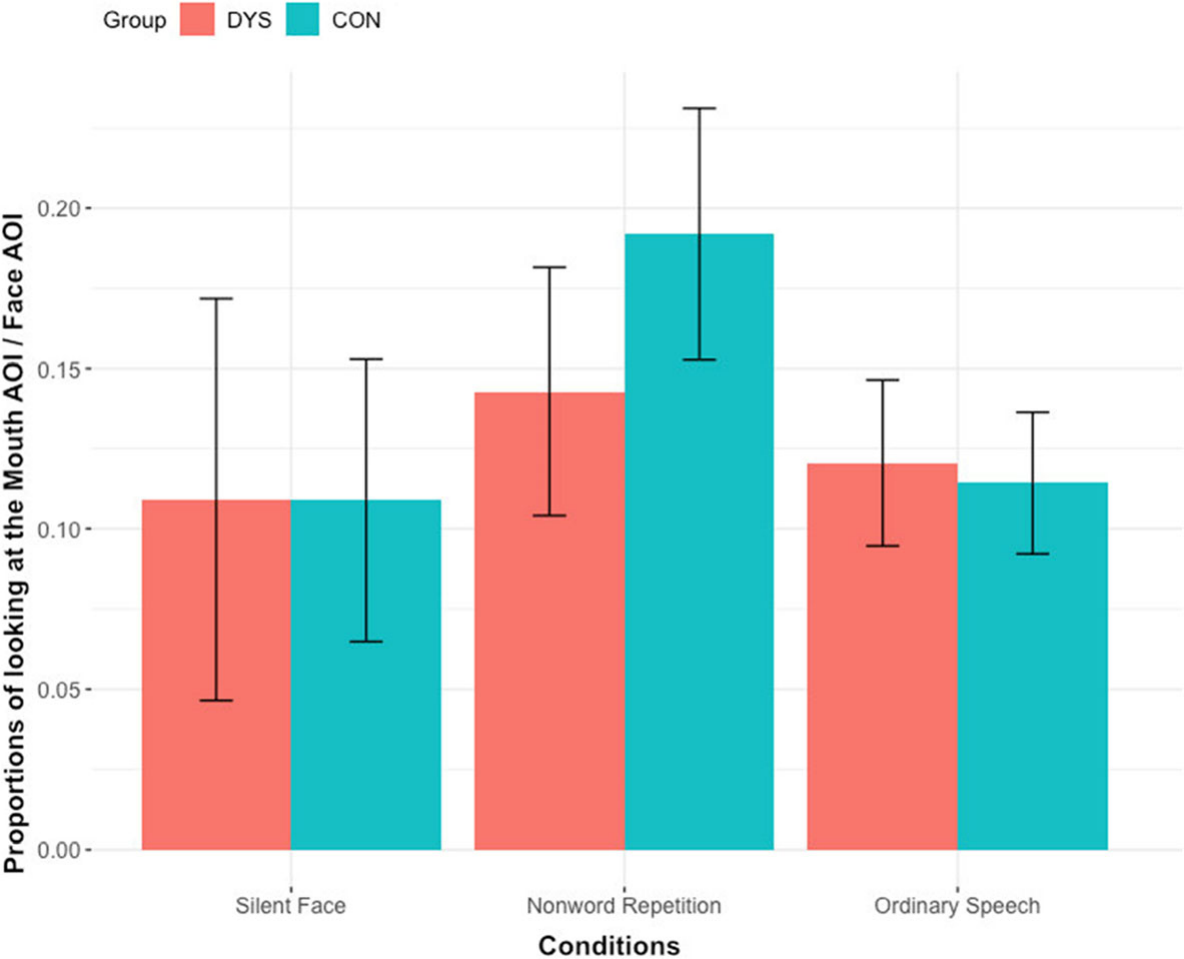
Mean proportion of fixation durations at the mouth AOI during the silent face, ordinary speech, and nonword repetition conditions in the DYS and CON groups. Error bars represent 99% CI

In order to examine how much children looked at the mouth while observing the speaker’s face, the duration of fixation to the mouth was calculated as the proportion of fixation duration to the mouth divided by the total fixation to the face (mouth AOI/face AOI; Fig. 1b) in the three facial speech conditions. A repeated measures ANOVA with condition (silent face, ordinary speech, nonword repetition) as a within-subject factor and group (DYS and CON) as a between-subject factor indicated a significant main effect of condition *F*(1.43, 57.24) = 5.27, *p* = 0.015, *η*^*2*^ = .116, Greenhouse-Geisser corrected, with a large effect size. Pairwise comparisons confirmed that, for the collapsed group, the proportion of looking at the mouth was higher during the nonword condition (*M* = .17, *SE* = .02) than when observing a silent face (*M* = .11, *SE* = .01; *p* = .017) or during ordinary speech (*M* = .12, *SE* = .01; *p* = .021). The analysis showed no main effect of group (*p* = .616) nor condition by group interaction (*p* = .275) though there seems to be a trend for a slight attenuation of mouth gaze in the dyslexia group in the nonword repetition condition (Fig. 2).

In order to further examine the relationship between reading and reading-related skill measures and the proportion of looking at the mouth during the three facial speech conditions, correlational analyses were conducted within each group.

For the dyslexia group, the Spearman correlational analyses indicated a moderate significant correlation between the time spent looking at the mouth during nonword repetition condition and LäSt word subscale, r*s*(17) = .494, *p* = .044, and at trend level, with LäSt nonword subscale r*s*(17) = .428, *p* = .087. There were no indications of an association with phonological awareness r*s*(17) = .086, *p* = . 74. The SDQ total score of traits related to comorbid psychopathology also correlated moderately but non-significantly with time spent looking at the mouth during nonword repetition in the dyslexia group, r*s*(17) = −.426, *p* = .08. Looking during the silent face and ordinary speech conditions did not correlate with any standardized measures related to reading, phonological awareness, or comorbid psychopathology in the dyslexia group. All p*s* were > .1 and .5, respectively.

For the control group, the correlations between proportion of looking at the mouth during nonword repetition task and scores on LäSt word subscale (r*s*(25) = .14, *p* = .488), phonological awareness (r*s*(25) = .109, *p* = .60), as well as the SDQ total score (r*s*(25) = .03, *p* = .89) were relatively weak and did not approach significance in any case. The correlation with LäSt nonword (r*s*(25) = .323, *p* = .116) was moderate but not significant. Much like in the DYS group, looking behavior during the silent face and ordinary speech conditions did not correlate with any standardized measures related to reading, phonological awareness, or comorbid psychopathology.

These results present a potentially interesting case for dyslexia, in particular: while the analysis did not show clear and significant diagnostic group differences in the proportion of looking at the mouth, correlational findings point to a positive association between mouth looking and reading skills, when examining reading ability dimensionally. This means that those individuals with a dyslexia diagnosis who are relatively more developed readers also are the ones who look at the mouth, specifically in the condition where the task was to decipher phonologically demanding speech. While these findings are suggestive of “mouth reliance” in at least some children with dyslexia, the question remains as to whether children with dyslexia also benefit from facial speech when processing written words. This is what we addressed in Study 2, where we examined whether children with dyslexia functionally benefit from visual presentation of facial speech when presented with grapheme-based decoding task.

## Study 2

In the second study, we examined whether the presence of articulation cues of the mouth during speech perception is functionally beneficial when confronted with a phoneme-/grapheme-based word decoding task in children with and without dyslexia. As both facial speech processing and phoneme/grapheme associations are fundamentally audiovisual processes (Francisco et al., 2018), and have been shown to partly share neural circuitry (Blomert, 2011), we were particularly interested in examining the possibility that the presence of an articulating mouth may enhance the quality of word encoding, by making phoneme/grapheme pairings clearer. Interestingly, the presence of articulation cues has been shown in prior experimental research to affect aspects of psycholinguistic processing, including facilitating upcoming word recognition (Hernández-Gutiérrez et al., 2018) and encoding during voice learning (Sheffert & Olson, 2004). For instance, in one study (Hernández-Gutiérrez et al., 2018), adults listened to short stories in which one target word was either expected from the story context or unexpected. While a late posterior positive ERP was observed in response to the expected target word, the effect was significantly reduced when the mouth was covered suggesting that the presence of the mouth may indeed enhance comprehension. To our knowledge, there is no prior research exploring the possibility that presenting phoneme/grapheme combinations together with facial speech might affect how well children with dyslexia are able to read this material.

In the context of children and reading development problems, several educational studies have, however, included training sessions that have focused on improving articulatory awareness. Articulatory awareness training can, for instance, include pairing of phonemes with graphemes, and it can also put the emphasis on the shape of the mouth associated with a particular sound it makes (the term “viseme” is commonly used to refer to the unique mouth shape that correspond to one or more phoneme). One study with pre-reading typically developing preschoolers found that pairing phonemes with visemes improved word reading when compared with presenting written letters (graphemes) alone (Boyer & Ehri, 2011). In another study (Fälth et al., 2017), pre-school children from the general population received over 2700 min of training pairing visemes and their corresponding sounds. The study reports positive results in reading ability and phonological awareness with long-term generalization to new words and speech sounds.

Previous findings are not, however, consistent. In particular, some studies showed no additional benefit in using articulatory awareness training for children with severe reading impairments/dyslexia. Building on training of phonological auditory discrimination (Lindawood & Lindawood, 1975), in one study (Wise et al., 1999), children used mirrors and utilized tactile information from their own faces (felt their faces with their hands) to discover articulatory movements that resulted in different sounds. In another study (Torgesen et al., 2001), poor readers were similarly presented with distinctive kinesthetic, auditory, and visual features associated with common phonemes. In these studies, however, there were no evidence of any added value of articulatory awareness training beyond phonological discrimination training in terms of reading outcomes. Critically, in both studies, the focus seems not mainly to be on the speaker’s mouth, but on the child’s own mouth, either by having them touch their own mouth (Wise et al., 1999) or visually inspecting their own mouth shape in a mirror (Torgesen et al., 2001), leaving unclear to what extent these studies actually address the issue of the importance in the use of observed articulatory *cues in others* while processing facial speech.

Moreover, while the large-scale long-term intervention studies described above are highly valuable from the perspective of informing educational practice, they often lack experimental control sufficient for exploring detailed causal relations between sensory and cognitive functions and patterns of learning. Given this, the current experiment had a much more immediate aim: directly testing whether access to articulatory speech movement during written word encoding improves word reading accuracy and fluency “on the fly” in children with and without dyslexia. In order to achieve this, we created a new computerized program in which children gazed at a screen, while a series of words were written and spelled out one at a time. We name this condition “phonic reading.” Half of the presented words were accompanied by a video presentation of a mouth pronouncing each phoneme. We then examined if the presence of the mouth pronouncing the words during encoding had an influence during independent (offline) reading of these same words.

We hypothesized that if children with dyslexia are insensitive or unable to utilize articulatory clues from the mouth (“mouth insensitive”), we would observe no difference in terms of accuracy and speed when reading the words presented with the mouth compared with those without. If, however, children with dyslexia rely heavily from the presence of the mouth in order to compensate for auditory-phonological problems (“mouth reliance”), we might expect them to perform better in accuracy and speed during reading words presented with the mouth, potentially through higher quality encoding during the presentation of the words in the facial speech condition.

### Method

#### Participants and ethics

This is the same as in Study 1.

#### Experimental procedure

At the start of the video, the participants were instructed to observe the screen and attend to the words being presented. Participants were shown 30 words, which were on average 2 syllables long (for a complete list, see Supplementary Materials). All words were presented in their written form one at a time in the middle of the screen. Each word was first presented in its entirety and then read phonetically by either a male or female speaker. As the speaker pronounced each phoneme, the corresponding letter was bolded (phonic reading). The word was then repeated in its entirety at the end. In half of the trials (15 words), the written word was also accompanied by a video of the speaker with only mouth visible (phonic reading + facial speech). Therefore, while all the words were presented with the phonemes being spelled out, the difference between the conditions was the presence or not of facial speech. Each child saw each word only once. The gender of the speaker and the condition was counterbalanced across participants, and the order of the word presentation was random. The average word length, number of syllables, and the number of unique visemes (Beskow, 1995 as cited in Engström, 2003) was similar between the two conditions (all *p*s > .7). The entire presentation lasted approx. 10 min.

**Fig. 3:**
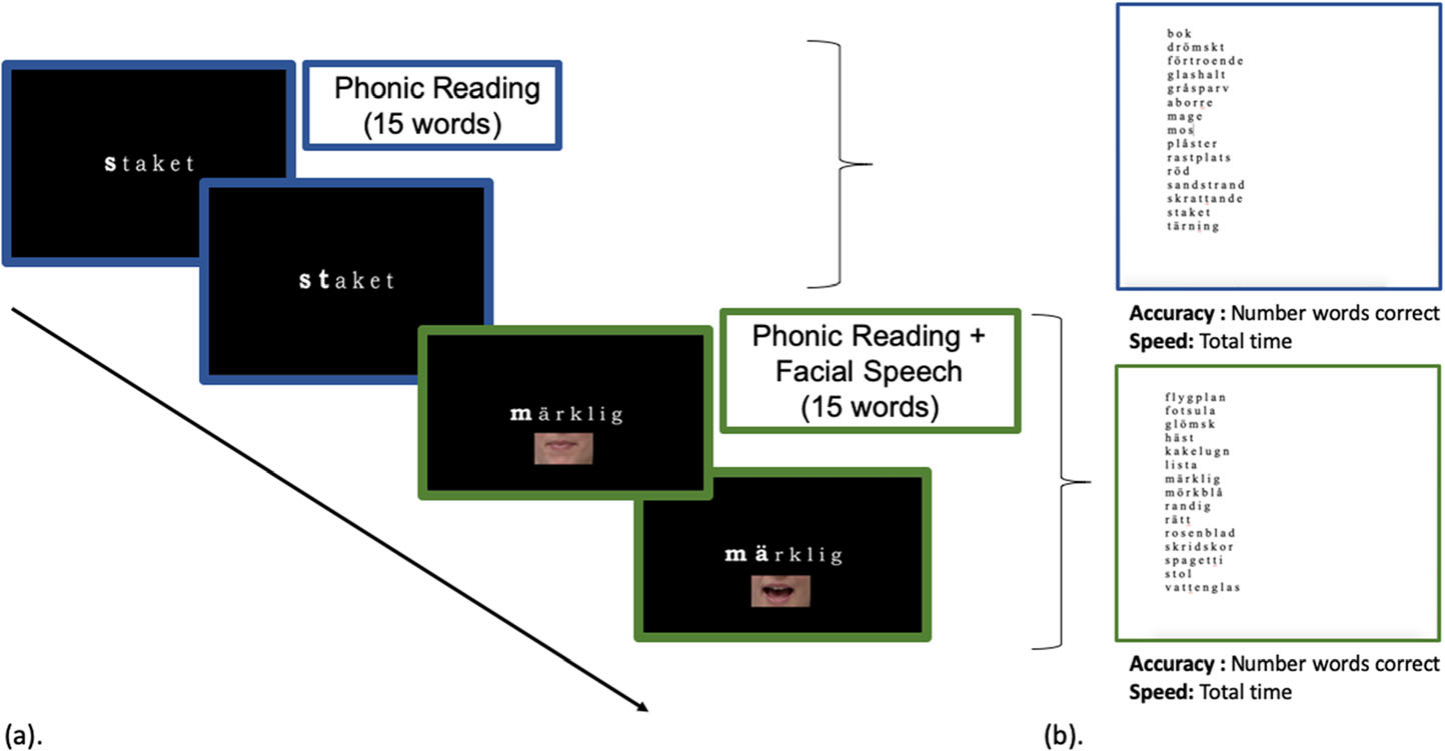
**a** Phonic reading and phonic reading + facial speech conditions and **b** the corresponding word lists in the offline reading task

After watching the video, the participants were presented with two lists of words, one at a time, written on a piece of paper. Each list contained 15 words that they just saw in the video. One list contained only those words that were presented in the phonic reading condition, and the other list corresponded to words in the phonic reading + facial speech condition which was presented with a speaker’s mouth (Fig. 3). The words on the list were in random order, and we counterbalanced across participants which words were shown with or without facial speech, as well as which list was read first. Participants were instructed to read the presented words as quickly and accurately as possible. Time it took to read the list started when the experimenter flipped the list over and ended when the participant finished reading the last word on the list. Reading accuracy was recorded.

### Data analysis

Data analysis procedure was the same as in Study 1. The exact parameters used for data analysis of Study 2 can be downloaded using uwid ts-aa9-872 from within the Time Studio program. Statistical analyses were performed using the package ggstatsplot2 (Patil, 2021) for the RStudio software environment (version 1.2.5033; RStudio Team, 2020).

### Statistical analysis

The main analysis examined the relative improvement in two outcome variables, speed and accuracy, following the presentation of the words in the two conditions.

*Speed* was calculated as the difference in time (T), measured in seconds, it took the child to read the list of words that had been presented with the mouth (phonic reading + facial speech) minus the time it took to read the list of words that had been presented without the mouth (phonic reading) such that:

$$ \Delta\ \mathrm{Speed}={\mathbf{T}}_{\left(\mathrm{Phonic}\ \mathrm{Reading}+\mathrm{Facial}\ \mathrm{Speech}\right)}-{\mathbf{T}}_{\left(\mathrm{Phonic}\ \mathrm{Reading}\right)} $$

A positive Δ Speed value indicates slower reading of the words presented with the mouth, while zero score indicates no change (Δ Speed).

*Accuracy* was calculated as the difference in the number of correct words read on the list presented in the phonics + facial speech condition minus phonics reading condition such that:

$$ \Delta\ \mathrm{Accuracy}={\mathbf{X}}_{\left(\mathrm{Phonic}+\mathrm{Facial}\ \mathrm{Speech}\right)}\hbox{--} {\mathbf{X}}_{\left(\mathrm{Phonic}\ \mathrm{Reading}\right)} $$

A positive value of the Δ Accuracy indicates more accurate reading of words when presented with the mouth.

The two outcome variables deviated from normality, and therefore group comparisons were carried out with non-parametric Mann-Whitney *U* tests, while correlation between Δ Accuracy, Δ Speed, reading ability (LäSt word and nonwords subscales), phonological awareness (NEPSY), and behavior (SDQ) in each group were examined using non-parametric Spearman correlations.

## Results and discussion

**Fig. 4:**
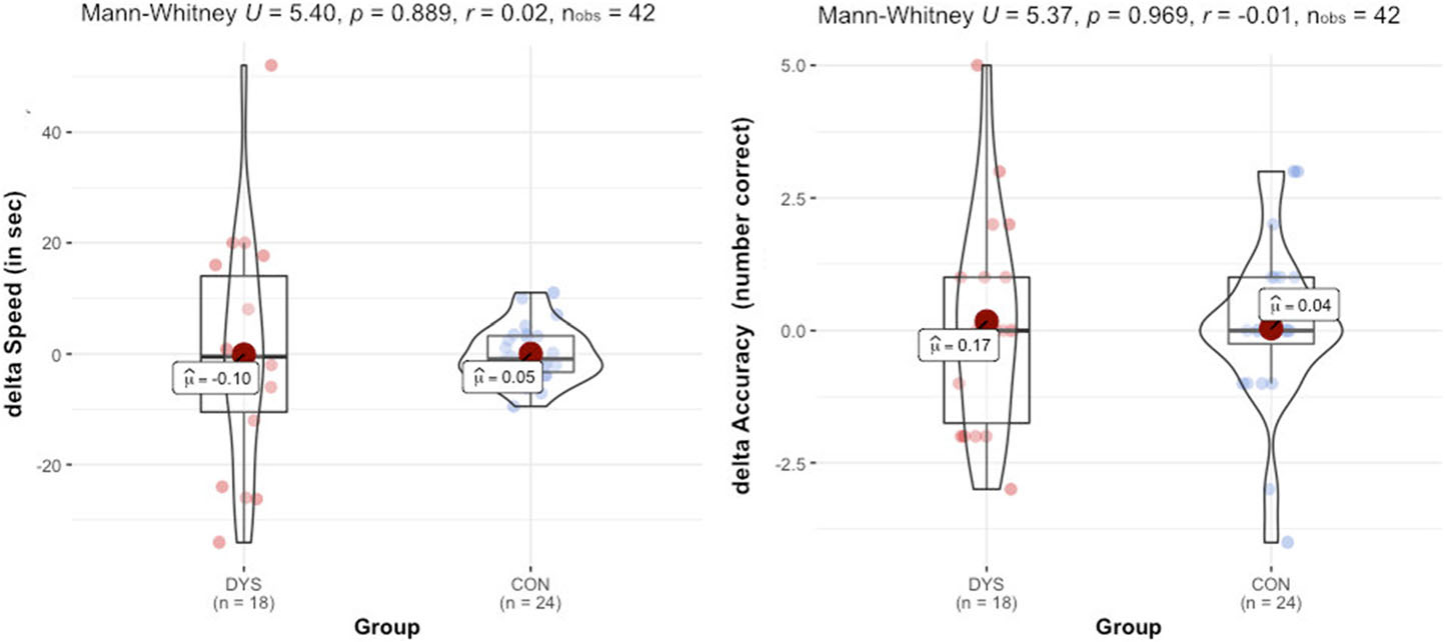
Group comparison of Δ Speed and Δ Accuracy scores. The title reports the Mann-Whitney statistic, the significance level, the effect size, and the number of observations

The between-group comparisons showed that both Δ Speed (*p* = 0.889) and Δ Accuracy (*p* = 0.969) did not differ between groups with very small effect size (*r* = .02 and −.01, respectively) (Fig. 4). Moreover, the mean scores are very close to zero, meaning that we found no evidence that the presence of facial speech had any effect on group performance in either group.

In order to examine individual differences within the groups, the relationship between Δ Accuracy, Δ Speed, and reading ability was examined. Spearman correlation analyses revealed positive large associations between Δ Accuracy and LäSt word reading scores r*s*(18) = .527, *p* = .024, and LäSt nonword reading scores r*s*(18) = .614, *p* = .007 for the DYS group. This means that those children in the DYS group who more accurately read words from the lists that had been presented with the mouth (phonic reading + facial speech) performed better on the standardized measures of reading ability. Accuracy was not associated with neither phonological awareness nor SDQ total score (both *p*s >.17). Δ Speed suggested possible trends with LäSt word subscale *r*s(18) = −.292, *p* = .239, LäSt nonword subscale *r*s(18) = −.428, *p* = .076, as well as phonological awareness score rs(18) = −.461, *p* = .054. A negative correlation with speed would have suggested that those who are better at reading and phonological awareness are faster at reading words encoded with a mouth (i.e., in the phonic reading + facial speech condition). We did, however, find that speed correlated significantly with SDQ total scores, r*s*(18) = .486, *p* = .041, suggesting that those children with dyslexia who display higher comorbid traits improved less in terms of reading speed of words encoded with facial speech.

For those in the CON group, there were no significant correlations between Δ Accuracy or Δ Speed and scores of reading ability or phonological awareness (all p*s* >.3). There were also no significant correlations in the CON group between Δ Accuracy or Δ Speed and SDQ total scores (both p*s* > .5).

**Fig. 5:**
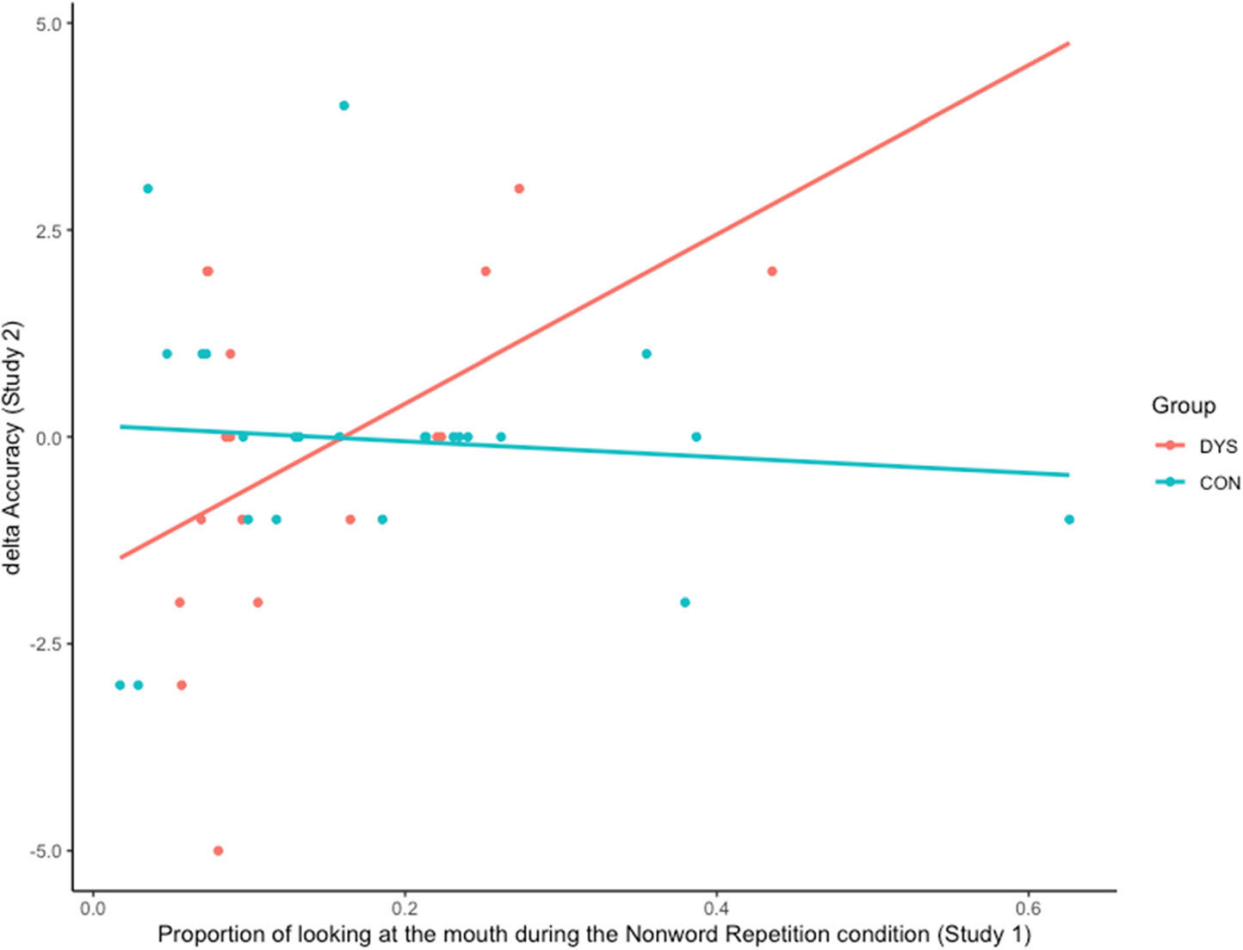
Scatterplot showing a correlation between proportion of mouth looking during the nonword repetition condition (Study 1) and the difference in accuracy of the number of correct words from lists presented in the phonic reading + facial speech condition and phonic reading-only condition (Study 2)

Motivated by these results, as well as our suggestive findings from Study 1 where we found moderate correlations between mouth looking and scores on reading ability, we next examined to what extent mouth gazing and Δ Accuracy associate with one another. That is, we examined whether looking toward the mouth during the nonword repetition condition (Study 1) was associated with the improvement in accuracy when presented with words augmented with facial speech information (phonic reading + facial speech; Study 2). Indeed, we found such a correlation (*r*s = .49, *p* < .05, Fig. 5) for the DYS group, but not for the CON. This finding suggests that some better reading children in the DYS group not only spontaneously orient toward the mouth during facial speech processing, but they also seem to benefit in terms of better reading accuracy of words encoded with facial speech cues.

## General discussion

The aim of the present study was to examine the role and functionality of facial speech (i.e., articulatory cues) during speech perception and word decoding in a group of school children with and without dyslexia. The current study is, to our knowledge, the first to use eye tracking technology to examine natural gaze behavior when observing silent and speaking human faces in developmental dyslexia. It is also the first study to experimentally look at the functional aspects of facial speech cues in reading performance.

Research has shown that when auditory information is difficult to process, adult listeners rely on visual information from the moving mouth to decipher speech (Driver, 1996). In the course of early development, children change in the way they spontaneously look at talking human faces as a function of language development (de Boisferon et al., 2018; Lewkowicz & Hansen-Tift, 2012). At the same time, increased and over-reliant attention on the mouth, beyond the period of early language acquisition, has been associated with atypical communication development that hallmarks disorders like autism and language impairment (Åsberg Johnels et al., 2014; Falck-Ytter et al., 2010; Habayeb et al., 2020; Hosozawa et al., 2012). Such insights have been made possible using eye tracking technology, and here we adopted this straightforward method to examine possible facial speech processing alterations in developmental dyslexia, an area of research characterized by mixed findings.

In Study 1, we examined spontaneous mouth looking across conditions that varied in speech difficulty. The logic behind the study design was that if part of the deficit in dyslexia is that children are not able to make use of the available facial speech information (such as the lips and mouth shape), they would not modulate gaze patterns across the conditions, regardless of linguistic processing demands. Indeed, in creating the nonword repetition condition, we aimed to challenge children enough to elicit attention to the mouth (if the child was able benefit from this), without making it so difficult so as to risk complete task disengagement. We found no interaction with group in the overall ANOVA, and children across groups tended to increase the proportion of time fixating at the mouth during the phonologically challenging (nonwords) condition than when observing a silent actor or one that was talking in ordinary speech. The tendency to look at the mouth during nonword condition was slightly higher in the non-dyslexic group, although the difference failed to reach statistical significance. This general pattern is consistent with previous findings (Barenholtz et al., 2016; Sumby & Pollock, 1954) which show that when processing demands increase, observers become more *reliant* on the visual information from the mouth area.

Critically important, however, were the possible insights gained from examining individual differences within the dyslexia group. Indeed, when considering the groups separately, we observed a moderate correlation between reading ability (LäSt scores) and the proportion of time spent fixating on the mouth during the nonword repetition condition in the group of children with dyslexia, but not in age- and listening comprehension-matched controls. Thus, we find that within children who meet the criteria for dyslexia but nonetheless score relatively higher on reading efficiency also tend to fixate proportionally more at the mouth during phonologically demanding tasks.

In Study 2, we further examined the influence of facial speech in reading ability, this time when facial speech cues were presented together with written words. Inspired by some positive findings from intervention studies on typically developing readers designed to improve articulatory awareness (Boyer & Ehri, 2011; Fälth et al., 2017), we presented both groups with words and with/without facial speech information and tested them on speed and accuracy of reading. There were no significant group differences based on the presentation condition in speed and accuracy during the offline reading test of the presented words. However, like in Study 1, correlational analysis revealed that within the dyslexia group, those who were more accurate on reading words presented with the mouth also scored higher on standardized measures of reading ability.

Correlational analyses also revealed that children diagnosed with dyslexia who attended more to the mouth in the nonword repetition condition (Study 1) made less errors when reading words that had been presented with facial speech cues (Study 2). This finding suggests that some better-compensated children with dyslexia both spontaneously rely on information from the mouth to decipher phonologically difficult speech and effectively use this type of information to support decoding.

It should be clearly acknowledged that these associations are just that: associations. This means that there could be confounding or moderating influences beyond the scope of our data. If there is a causal relation between mouth gaze patterns, reading skills, and reading improvement with facial speech in dyslexia, we do not yet know the direction of such an influence. Also, other (third variable) factors can potentially affect the correlations. For instance, in our study, we found that individual differences in comorbid (psychopathological) traits in the dyslexia group were associated with one of the outcome variables, namely with reduced benefit in reading speed with added facial speech information. Future research is needed to determine the robustness, the mechanisms, and possible causalities underlying all these associations. Furthermore, because the present study is novel in several regards, more studies with similar setups or direct replication with larger samples are imperative.

Considering the findings across the two studies in light of the “mouth insensitivity” and “mouth reliance” hypotheses discussed in the Introduction, we find our results non-conclusive and that neither of these two theories can completely explain our findings. On the one hand, we cannot claim that when presented with a speaking mouth, all dyslexic children tend to disproportionately look at it. On the other hand, it is not accurate to say that dyslexic children are completely insensitive to the presence of a speaking mouth. Rather, it seems that, much like what is observed in controls, gazing toward the mouth is contextual and depends on task difficulty (Study 1) as well as each individual person’s learned tendency. That is, while not all dyslexic children look at the mouth in order to decipher difficult speech, those who do are better readers and also tend to benefit from mouth watching when learning to decode words (Study 2). Given what we know from training studies, it is possible that directing visual attention to the mouth can improve reading ability (Boyer & Ehri, 2011; Fälth et al., 2017), but perhaps unless it is part of the training protocol, only some, but not all children with dyslexia, will spontaneously do so. One practical implication in improving the efficacy of training studies would therefore be to determine how a particular child naturally looks at a speaking face. Identifying those children who spend very limited amount of time attending to these articulatory cues might help identifying an important determinant of the individual child’s treatment gains, a good example of precision medicine. Another interesting approach would be to monitor possible changes in natural face scanning patterns, while children with dyslexia take part in intense training that focuses on articulation and phonological awareness (e.g., Fälth et al., 2017). A critical task for future research is thus to understand the nature of the association suggested here between mouth gazing and reading skills in dyslexia. Specifically, it is important to determine the directionality of the association: do better readers look more to the mouth or are those who tend to make use of presented information from the mouth developing to become better readers? In order to address this, longitudinal studies, as advocated by Goswami (2015), would be helpful.

Finally, it is important to discuss certain limitations of the present study. One is the number of participants. Although the sample size is similar, or even larger, than in previous comparable research on facial speech processing in dyslexia (e.g., Rüsseler et al., 2018; Schaadt et al., 2016), it is fully possible that with even greater number of participants, and increased statistical power, more subtle differences between groups would be statistically apparent. That would also allow for a more robust exploration of mediating and moderating effects such as those of age, gender, general cognitive ability, and comorbidity with other neurodevelopmental or psychopathological conditions. We hope that further research will confirm—or challenge—our observed effects.

A second limitation pertains to the tasks and experiments. Indeed, when developing the eye tracking test battery used in the current study, we made several methodological choices whose relevance could not be predicted from our knowledge at the time. For instance, we still do not know to what extent the increased mouth looking in the nonword repetition condition may be due to the fact that in only this condition, the participants had to effectively repeat the words rather than only passively observe and listen, introducing potential motivational differences between conditions. Expanding the number of tasks, such as including tasks in the other conditions as well, will address this limitation in future studies. Nevertheless, despite these existing caveats, our results do show the potential in the use of eye tracking technology to provide insight into facial speech processing in dyslexia and to offer better understanding of potential treatment outcomes, while clearly pointing to the importance of individual differences in this “group.”

## Supplementary information

ESM 1 (DOCX 62 kb)

## Footnotes

1 One child did not have a formal diagnosis; however, this child already has received support for dyslexia in her school. During the assessment, which was carried out by the senior author who is a qualified dyslexia assessor, she displayed poor word and nonword reading skills, while her listening comprehension was in the normal range. She had no hearing or visual impairments according to parent questionnaire.
