# Supplementary material for: Facial speech processing in children with and without dyslexia
Source: Ann Dyslexia. 2021 Jun 11;71(3):501–24. doi: 10.1007/s11881-021-00231-3 (PMC8458188; doi:10.1007/s11881-021-00231-3)
Supplement: Supplementary file 1 — (DOCX 62 kb) [file 11881_2021_231_MOESM1_ESM.docx]

**Facial speech processing in children with and without dyslexia**

Supplementary

**Is there a difference in the amount of time spent looking at the mouth in the Phonics + Facial speech condition in Study 2?**

We examined the amount of time spent on the mouth by each group. Fixation durations within the Mouth AOI during the Phonic Reading + Facial Speech condition were calculated as the proportion of total fixation duration in the Mouth AOI and the sum of total fixations at the mouth and the word (Mouth AOI / (Mouth AOI + Word AOI)). Mouth AOI consisted of a square area marked around the image of the speaking mouth, 400 pixels x 200 pixels (6.8 horizontal x 3.44 vertical visual degrees), and the Word AOI, 1920 pixels x 150 pixels (32 horizontal x 2.5 vertical visual degrees) was a rectangular strip across the screen encompassing the written word (Figure 1a). In order to test our hypothesis, we examined the proportion of time spent looking at the mouth for each of the two groups. We found no significant differences in the proportion of time fixating on the mouth between groups, *p* = .949 (Figure 1b).

(a).


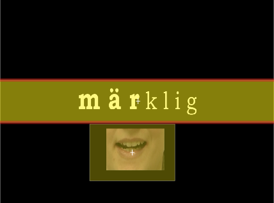


(b).
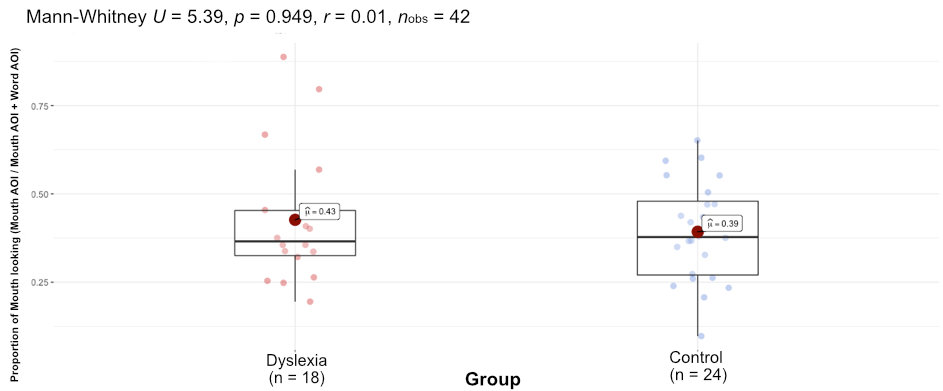


**Figure 1.** (a). Word AOI and Mouth AOI in the Phonic Reading + Facial Speech condition and (b). Boxplot illustrating the proportion of fixations to the Mouth AOI in the Phonics + Facial speech Condition in DYS and CON groups. The title reports the Mann-Whitney statistic, the significance level, the effect size and the number of observations.

**Nonword repetition (Study 1)**

s ä l i

l y k e

m å l e

r i p p e s a p p

v u l l e s å

m i n d e r k r a p s

l a n d o m a s t a

a l l o s i t t i

g i n d e r l i n d i

**Stories (Study 1)**

*Det var en gång en katt som blev jagad av en hund. Katten sprang ner för trappan och gömde sig under den. Då kunde inte hunden hitta katten.*

**Translation:**
There was once a cat that was chased by a dog. The cat ran down the stairs and hid under it. Then the dog could not find the cat.

*Det var en gång en flicka som var ute och åkte skridskor på en sjö. Plötsligt stannade hon till när hon såg att isen var för tunn. Hon vände tillbaka till stranden.*

**Translation:**
There was once a girl who was out skating on a lake. Suddenly she stopped when she saw that the ice was too thin. She returned to the beach.

*Det var en gång en häst som bodde i en hage på landet. Även om han trivdes där så längtade han efter att få springa fri. En dag hoppade han därför över stängslet och sprang iväg*.

**Translation:**

There was once a horse that lived in a paddock in the country. Even though he enjoyed himself there, he longed to run free. So one day he jumped over the fence and ran away.

*Det var en gång en man som bodde helt ensam i skogen. En dag började han längta efter andra människor. Han bjöd därför in folk från när och fjärran till en stor fest.*

**Translation:**

There was once a man who lived all alone in the woods. One day he began to long for other people. He therefore invited people from near and far to a big party.

*Det var en gång en pojke som hade sommarlov. Han var på stranden och blev sugen på en glass. Men precis när han köpt glassen så tappade han den i sanden.*

**Translation:**

There was once a boy who had summer vacation. He was on the beach and got hungry for an ice cream. But just when he bought the ice cream, he dropped it in the sand.

*Det var en gång en kvinna som körde en stor lastbil. Plötsligt så låg det ett stort träd över hela vägen. Kvinnan fick tvärbromsa lastbilen och hann precis stanna.*

**Translation:**

There was once a woman who drove a large truck. Suddenly there was a big tree across the road. The woman had to brake the truck and just had time to stop.

**Phonics reading and Phonics + Facial speech (Study 2)**

1. f l y g p l a n
2. f o t s u l a
3. g l ö m s k
4. h ä s t
5. k a k e l u g n
6. l i s t a
7. m ä r k l i g
8. m ö r k b l å
9. r a n d i g
10. r ä t t
11. r o s e n b l a d
12. s k r i d s k o r
13. s p a g e t t i
14. s t o l
15. v a t t e n g l a s
16. a b o r r e
17. b o k
18. d r ö m s k t
19. f ö r t r o e n d e
20. g l a s h a l t
21. g r å s p a r v
22. m a g e
23. m o s
24. p l å s t e r
25. r a s t p l a t s
26. r ö d
27. s a n d s t r a n d
28. s k r a t t a n d e
29. s t a k e t
30. t ä r n i n g
